# Observer Variability in BI-RADS Ultrasound Features and Its Influence on Computer-Aided Diagnosis of Breast Masses

**DOI:** 10.4236/abcr.2015.41001

**Published:** 2015-01-09

**Authors:** Laith R. Sultan, Ghizlane Bouzghar, Benjamin J. Levenback, Nauroze A. Faizi, Santosh S. Venkatesh, Emily F. Conant, Chandra M. Sehgal

**Affiliations:** 1 Department of Radiology, University of Pennsylvania, Philadelphia, USA; 2 Department of Electrical Engineering, University of Pennsylvania, Philadelphia, USA

**Keywords:** Breast Imaging, Breast Cancer, Observer Variability, Computer-Aided Diagnosis

## Abstract

**Objective:**

Computer classification of sonographic BI-RADS features can aid differentiation of the malignant and benign masses. However, the variability in the diagnosis due to the differences in the observed features between the observations is not known. The goal of this study is to measure the variation in sonographic features between multiple observations and determine the effect of features variation on computer-aided diagnosis of the breast masses.

**Materials and Methods:**

Ultrasound images of biopsy proven solid breast masses were analyzed in three independent observations for BI-RADS sonographic features. The BI-RADS features from each observation were used with Bayes classifier to determine probability of malignancy. The observer agreement in the sonographic features was measured by kappa coefficient and the difference in the diagnostic performances between observations was determined by the area under the ROC curve, A_z_, and interclass correlation coefficient.

**Results:**

While some features were repeatedly observed, *κ* = 0.95, other showed a significant variation, *κ* = 0.16. For all features, combined intra-observer agreement was substantial, *κ* = 0.77. The agreement, however, decreased steadily to 0.66 and 0.56 as time between the observations increased from 1 to 2 and 3 months, respectively. Despite the variation in features between observations the probabilities of malignancy estimates from Bayes classifier were robust and consistently yielded same level of diagnostic performance, A_z_ was 0.772 – 0.817 for sonographic features alone and 0.828 – 0.849 for sonographic features and age combined. The difference in the performance, ΔA_z_, between the observations for the two groups was small (0.003 – 0.044) and was not statistically significant (p < 0.05). Interclass correlation coefficient for the observations was 0.822 (CI: 0.787 – 0.853) for BI-RADS sonographic features alone and for those combined with age was 0.833 (CI: 0.800 – 0.862).

**Conclusion:**

Despite the differences in the BI- RADS sonographic features between different observations, the diagnostic performance of computer-aided analysis for differentiating breast masses did not change. Through continual retraining, the computer-aided analysis provides consistent diagnostic performance independent of the variations in the observed sonographic features.

## 1. Introduction

Despite major advances in diagnostic breast cancer imaging, the yield for biopsying a breast lesion is still low and up to 85% of biopsies are found to be benign [1]. There continues to be a need for further innovations to improve confidence and reliability of breast imaging. In this context, several studies have proposed the use of computer algorithms and machine learning methods to improve the diagnostic value of breast ultrasound [2]-[7]. These computer based systems can serve as a second reader to decrease false positive rates of breast images [2]. In our earlier study, we introduced an approach that combines individual sonographic features quantitatively by machine learning to determine the probability of malignancy of solid breast masses [7]. The results show that the Bayesian method of weighting provides a systematic approach for combining ultrasound BI-RADS features yielding a high level of diagnostic performance, with an A_z_ of approximately 0.884. While the results are encouraging, variability in the diagnostic performance on repeated assessments is not known. The goal of this study was to determine the extent of variation in the computer-aided diagnosis between repeated interpretations of the breast ultrasound images. In brief, the variability in the diagnosis can result from two factors: 1) differences in feature selection and 2) differences in weighting of the individual features contributing to overall estimate of the probability of malignancy. In this study we investigate the role of both the factors. First, the observer variability in feature selection from three observations of the ultrasound images was measured by inter-rater kappa statistics. Second, the sonographic features from each observation were combined using Bayes model to determine the probability of malignancy. The diagnostic performances of the probability estimates of three observations were compared to determine diagnostic variability. Since the predictive values of the sonographic features are influenced by the age of the patients [7], we also evaluated the diagnostic performance of the sonographic features in conjunction with the patient age.

## 2. Materials and Methods

### 2.1. Image Acquisition and Analysis

This retrospective study was approved by institutional Review Board. 264 masses were obtained from 248 female patients with biopsy-proven solid masses and known mammographic BI-RADS. Sonographic images were acquired using broadband 12 - 5 MHz transducer and a Philips ATL 5000 scanner. 5 to 7 B-Scan ultrasound images including color Doppler were acquired per patient in radial and anti-radial planes.

Images were analyzed using the ACR BI-RADS ultrasound lexicon [8]. According to this lexicon, sonographic features of a solid breast mass [9] are grouped into shape, orientation, margin, lesion boundary, echo pattern, and posterior acoustic features. The observer with three-years prior training in general radiology underwent a self study session of the BI-RADS lexicon descriptors and of the training cases of breast images with known BI-RADS and pathology. The observer was blinded to patient age, race, physical examination, family history, mammographic report, and histological diagnosis during analysis.

The BI-RADS features assessment was repeated two more times after the initial assessment. The second observation (observation 2) was one month from the initial observation (observation 1) and the third observation (observation 3) was three months later. In all three observations the same image data was analyzed where the cases were presented to the observer in a random order.

Agreement in the BI-RADS features was determined by kappa statistics which assesses the inter-rater agreement beyond that is expected by chance [10]. According to this approach, *κ* = 1 corresponds to complete agreement whereas *κ* = 0 represents an agreement comparable to chance. The intermediate values between 0 and 1 represent the degree of agreement. On a five scale system described by Landis and Koch [11], kappa values 0.01 – 0.20, 0.21 – 0.40, 0.41 – 0.60, 0.61 – 0.80 and 0.81 – 1.00 were designated to indicate slight, fair, moderate, substantial, and almost perfect agreement, respectively. Both individual features agreement values and all features combined (overall) agreement values were calculated.

### 2.2. Computer-Aided Analysis

The sonographic BI-RADS features were used with machine learning algorithm to determine probability of malignancy. This involved training the algorithm using cases with known features and diagnosis. Following the training the algorithm was tested on the unknown cases to predict the probability of malignancy. The predicted values were compared with the biopsy results. The training and testing were performed by using leave-one-sample out cross validation. This involved training the algorithm on all cases of the database except one and predicting the outcome of the remaining last case. The process of training and testing was repeated recursively until the entire dataset has been analyzed. Training and testing was performed by using Bayes model in which the probability of an event (malignancy) is revised based on the accumulation of new evidence (detection of sonographic features). Bayes probability of malignancy in the presence of sonographic features P(M|F) was determined by the approach described earlier [12]. In short, it was determined by multiplying initial estimate of probability P(M) with the probabilities that feature F_i_ is present in the malignant mass P(F_i_|M). P(F_i_|M) was determined by dividing the ratio of number of malignant cases with feature F_i_ over the total number of malignant cases. P(M) was determined by the ratio of number of malignant cases to the total number of cases studied. The diagnostic performance of the Bayes probabilities P(M|F) was measured by calculating the area under the ROC curve (A_z_), the standard error, and the 95% confidence intervals [MedCalc Software, Ostend, Belgium].

The statistical difference between the diagnostic performances of the three observations was determined based on p-values [13]. A p-value less than 0.05 was considered to be statistical significant. Additionally, interclass correlation coefficients of the probability estimates were calculated as a measure of the consistency of the diagnostic performance in the three observations.

## 3. Results

### 3.1. General Characteristics

Of the 264 lesions, 85 (32%) were malignant and 179 (68%) were benign. Among the malignant lesions, invasive ductal carcinoma was the most common 65 (76%). Other diagnoses included invasive lobular carcinoma 7 (8%), ductal carcinoma in situ 7 (8%) including one papillary carcinoma in situ case, adenocarcinoma 3 (3%), two poorly differentiated carcinomas and one remaining case which was diagnosed as mucinous mammary carcinoma (a rare form of invasive ductal carcinoma). Of the benign masses, 44% were found to be fibroadenomas, 33% were identified as miscellaneous fibrocystic changes, 6% were sclerosing adenosis, and the remaining 17% were identified as benign lesions without atypia in the histopathology report. The mean (±standard deviation) age of all the patient population was 51.5 ± 14.7 years. The mean age of patients with malignant masses was 58.8 ± 12.1 years compared to 48.0 ± 14.5 years for benign cases. The difference in the mean age of the two groups was statistically significant (p = 0.0001).

### 3.2. Agreement in BI-RADS Feature Selection

**Figure 1.**
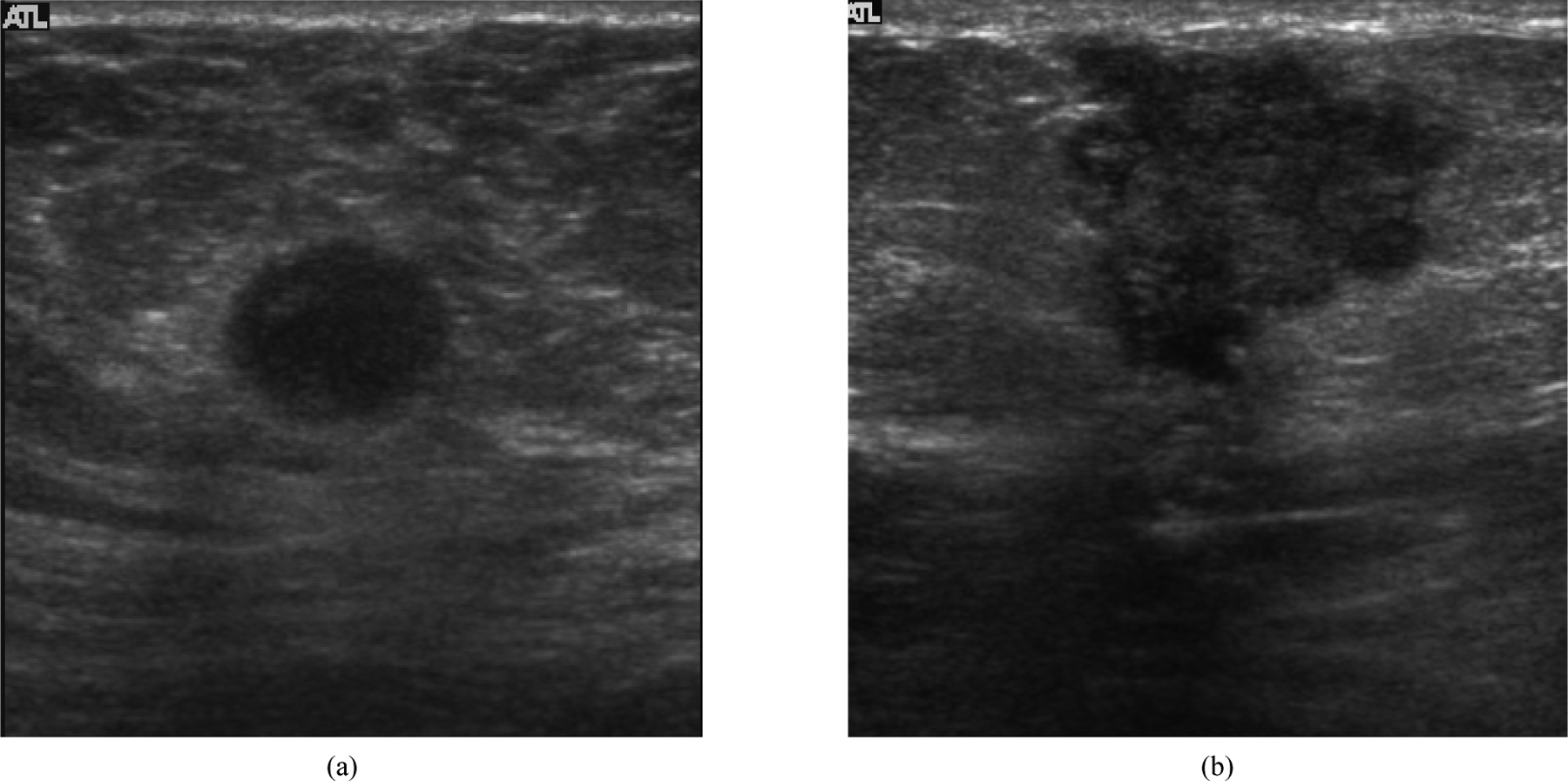
(a) Example of a breast mass that showed high agreement in sonographic features selected between the three observations; (b) Example of a breast lesion that showed lowest agreement in features selected over the three observations.

Figure 1 shows examples of two breast lesions with high and low agreement in feature selection between three observations. Features like oval shape, microlobulation and hypoechogencity were consistently observed in all three readings in the image shown in Figure 1(a). On the other hand, considerable variation in lesion orientation and margin features was observed between observations in the image shown in Figure 1(b). The results on agreement for each BI-RADS feature for all the cases are summarized in Table 1. *κ* for the individual features ranged from 0.16 to 0.95. The highest intra-observer agreement was found to be on the lesion echo pattern with *κ* between 0.69 and 0.98 for the three observations. The feature which showed the lowest agreement value was lesion boundary with *κ* between 0.15 and 0.53.

When all the features were investigated collectively, the overall intra-observer agreement between observations 1 and 2 made at an interval of 1 month was 0.77. *κ* for the agreement between observations 2 and 3 made at a time interval of 2 months was 0.66. For the time interval of 3 months between observations (observation 1 and observation 3) the agreement reduced to 0.56. Thus there was a progressive decrease in agreement (*κ*) as the time interval between the observations increased from 1 month to 3 months (Table 1).

### 3.3. Diagnostic Performance Analysis

**Figure 2.**
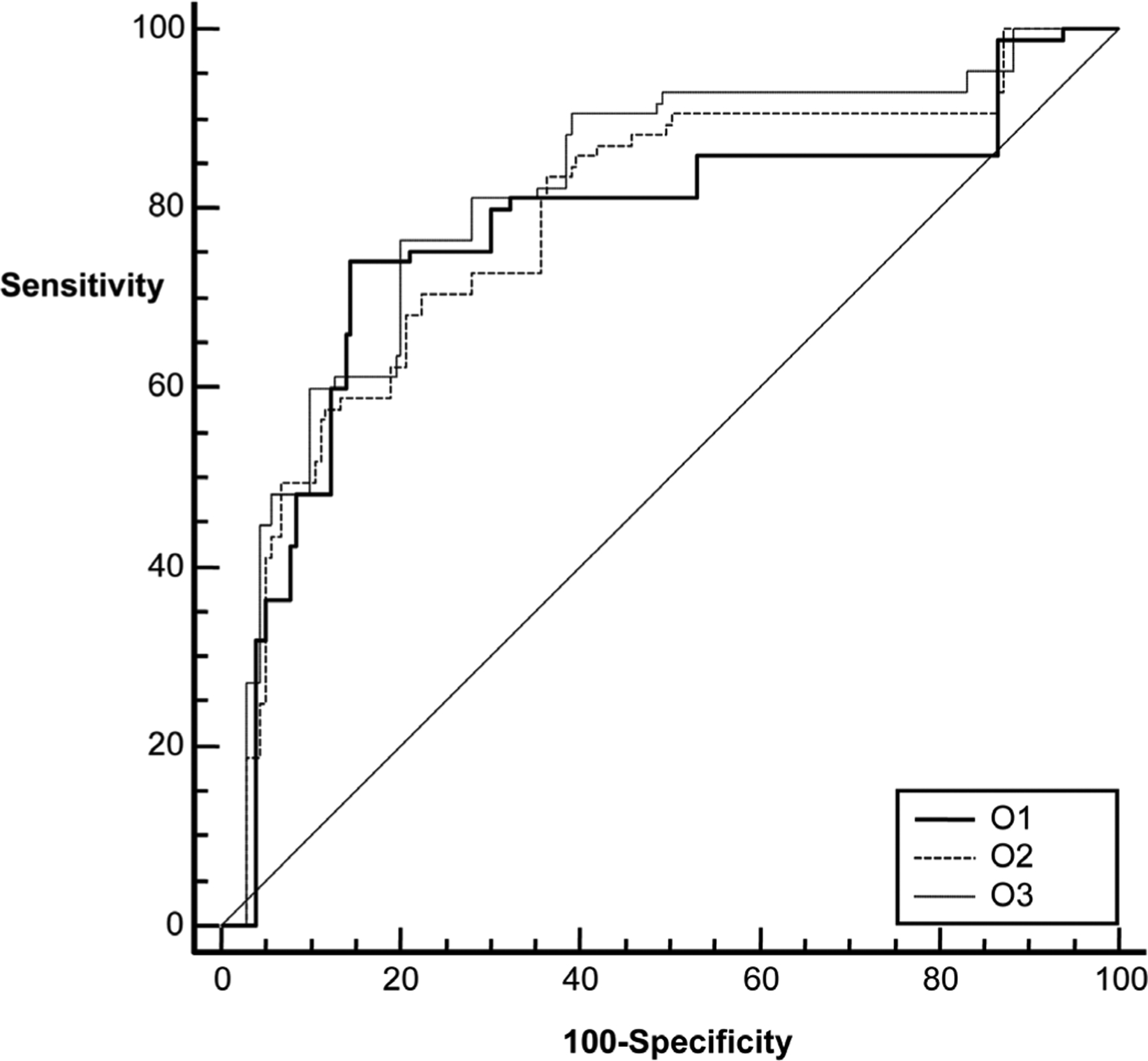
The diagnostic performances of Bayes probabilities estimates from three observations. O1, O2 and O3 refer to first, second and third observations, respectively.

**Figure 3.**
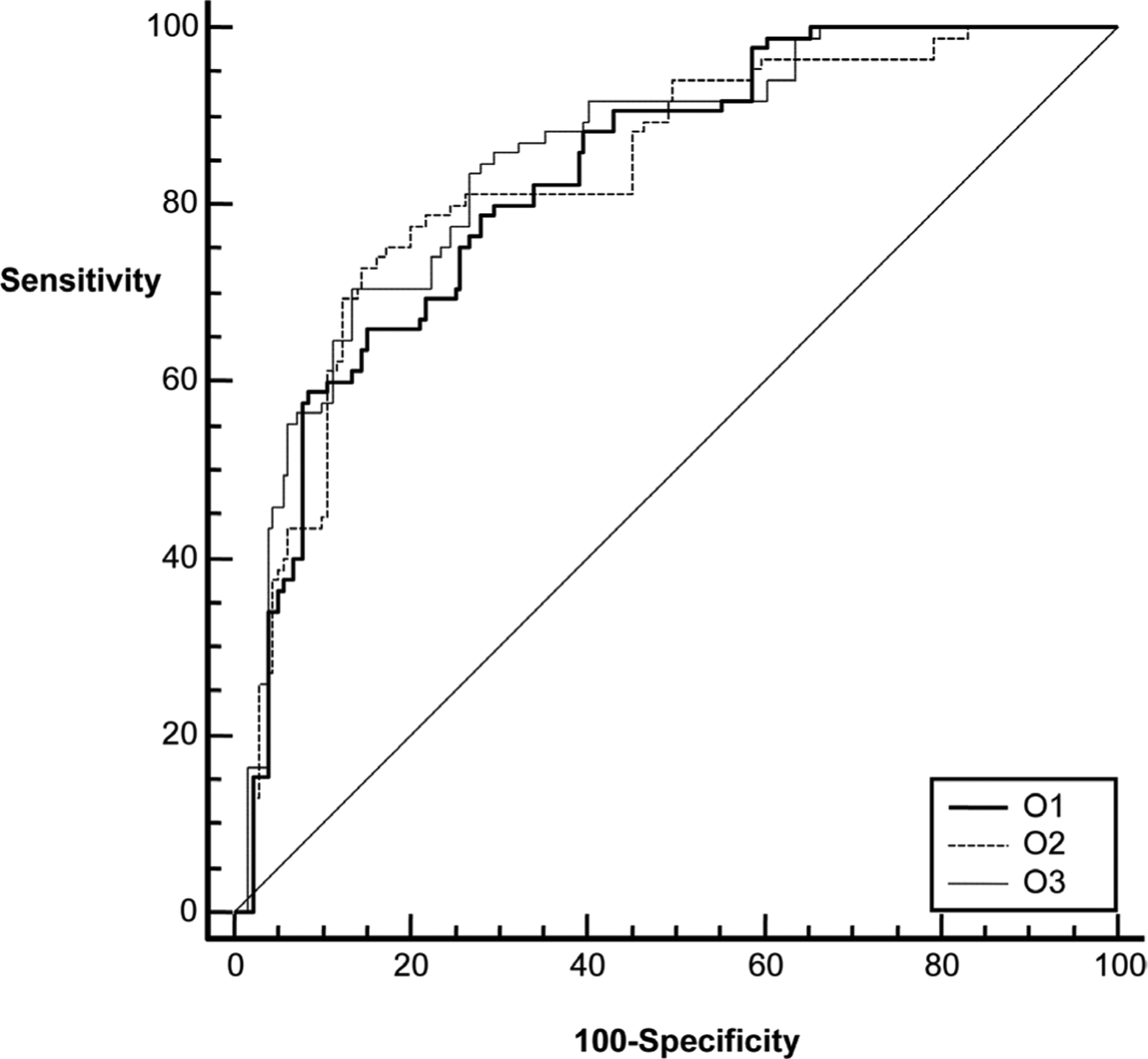
The diagnostic performances of Bayes probabilities estimates from three observations combined with patients’ age. O1, O2 and O3 refer to first, second and third observations, respectively.

The area under the ROC curve for the ultrasound features alone ranged from 0.772 to 0.817 for the three observations (Table 2 and Figure 2). The difference in the performance (ΔA_z_) between the observations was small (0.013 to 0.044) and not statistically significant (p > 0.05, Table 2). The diagnostic performance increased markedly (range: 0.828 – 0.849, Table 3 and Figure 3) when the age was included as a risk factor in estimating probability of malignancy. Similar to sonographic features alone, ΔA_z_ for sonographic features plus age was small (0.003 – 0.021, Table 3) and not statistically significant. Inter class correlation coefficient for the three observations was 0.822 (95% CI 0.787 – 0.853) for features alone and 0.833 (95% CI 0.800 – 0.862) for BI-RADS features combined with age.

## 4. Discussion

Previous studies evaluating the observer variability in the interpretation of BI-RADS sonographic features have shown that the agreement between observers can be fair to substantial [14]-[17]. Abdulla *et al*. [14], for instance, demonstrated that inter-observer variability as measured by kappa statistics (*κ*) for individual features ranged from fair (*κ* = 0.36) to substantial (*κ* = 0.70). Similarly, Calasa *et al*. [15] demonstrated that intra-observer variability for individual features ranged from moderate (*κ* = 0.59) to substantial (*κ* = 0.85) with an overall substantial agreement with kappa values ranging from 0.72 to 0.79. In general, variation in features observed in this study is comparable to the previously reported values, although the range of *κ* for individual features in the present study is wider (0.16 – 0.95).

The results of this study also show that the time interval between observations influences observer agreement and there is a steady decrease in *κ* with time between the observations. The reason for the steady decrease is not completely understood but could be potentially due to the “recall effect” described by Ryan *et al*., when reviewing the same chest X-ray image repeatedly [18]. When the observations are made close together in time, the user is influenced by the memory of earlier observation, thus creating an unconscious recall bias. As the time between the observations increases, the influence of the earlier observations becomes less pronounced, thus reducing agreement. The results demonstrating a change in agreement with time have not been previously reported and they suggest that the time interval between the observations must be controlled in designing observer agreement studies.

Prior studies evaluating the variability in breast cancer diagnosis with ultrasound have primarily focused on the variability caused by feature selection. While useful, this assessment alone is not complete because the process of diagnostic assessment of a breast lesion is a two-step process where feature selection is followed by weighting of the features to determine the combined probabilities of malignancies. The previous approaches did not take into consideration how the second step of weighting the individual features contributes to observer variability in diagnostic performance. The results of this study show that despite the variability in the individual feature between the three observations, the final diagnostic performances are comparable. These results are further supported by a strong interclass correlation between the probability estimates approaching 0.83. Although there was a notable variation in individual sonographic features between observations, the diagnostic performances did not change. The seeming discrepancy between observations is not surprising because the computer system is trained on the observed features, thus it is able to discount the differences in feature selection by weighting them differently toward assessing probability of malignancies. In essence, the continuous retraining of the computer system on the observed features compensates for the variation in feature selection. Although this study used Bayesian classifiers for computer aided diagnosis, it is reasonable to anticipate that similar patterns should holds for other learning algorithm. It is also conceivable that individual observers may compensate for the variations in features detection by weighting them differently towards the final diagnosis between observations. Thus, the future studies evaluating diagnostic variations between observations should go beyond studying variations in individual BI-RADS features only; they should also include assessment of the diagnostic performances. Although the results presented in this study are encouraging and demonstrate the efficacy of BI-RADS, further studies with multiple readers are needed for a comprehensive understanding of observer variability in breast ultrasound.

In conclusion, ultrasound images of breast masses were analyzed repeatedly using BI-RADS lexicon. When the features were considered together as a group, the observer agreement was moderate to substantial. However, there were notable differences when features were compared individually. Despite differences in the individual sonographic features between readings, the diagnostic performance of computer-aided analysis of malignant and benign breast masses did not change. Through a built-in learning process in the algorithm, the computer-based analysis was able to account for feature variations and thus provided an effective method to differentiate malignant and benign breast masses.

## Figures and Tables

**Table 1.** Intra-observer agreement values for BI-RADS US descriptors. The term “overall” represents agreement in all the features together. O1, O2, and O3 refer to first, second and third observations respectively.

Feature	O1 vs. O2 (1 month interval) (*κ*)	O2 vs. O3 (2 months interval) (*κ*)	O3 vs. O1 (3 months interval) (*κ*)	Intra-observer (*κ*) [15]	Intra-observer (*κ*) [16]
Shape	0.51	0.75	0.46	0.71	0.73
Orientation	0.65	0.71	0.56	0.83	0.68
Boundary	0.16	0.53	0.15	0.85	0.68
Echo pattern	0.98	0.70	0.69	0.67	0.65
Posterior acoustic features	0.98	0.69	0.67	0.82	0.64
Margin	0.95	0.56	0.56	0.59	0.64
**Overall**	**0.77 (Substantial)**	**0.66 (Substantial)**	**0.56 (Moderate)**	**0.77 (Substantial)**	**0.74 (Substantial)**

**Table 2.** Area under the ROC curve (A_z_), the standard error (SE), 95% confidence interval (95% CI) and the p-value for Baysian estimated probabilities in the three observations. Observation 1 represents the initial observation. Observations 2 and 3 were made 1 and 2 months after observation 1.

	A_z_ ± SE	95% CI	ΔA_z_ and p-value		
**Observation 1**	0.772 ± 0.35	0.717 – 0.822	p = 0.49 **Δ**A_z_ = 0.013		p = 0.09 **Δ**A_z_ = 0.031
**Observation 2**	0.786 ± 0.32	0.731 – 0.834		p = 0.08 **Δ**A_z_ = 0.044	
**Observation 3**	0.817 ± 0.029	0.765 – 0.862			

**Table 3.** Area under the ROC curve (A_z_), the standard error (SE), 95% confidence interval (95% CI) and the p-value for Baysian estimated probabilities combined with patient age in the three observations. Observation 1 represents the initial observation. Observations 2 and 3 were made 1 and 2 months after Observation 1.

	A_z_ ± SE	95% CI		ΔA_z_ and p-value	
**Observation 1**	0.828 ± 0.0258	0.777 – 0.872	p = 0.87 ΔA_z_ = 0.003		p = 0.17 ΔAz = 0.021
**Observation 2**	0.831 ± 0.027	0.780 – 0.874		p = 0.39 ΔA_z_ = 0.012	
**Observation 3**	0.849 ± 0.0248	0.800 – 0.890			
